# Retraction Note: Acute coronary syndrome due to left main coronary trunk compression 2 months after left atrial auricle clipping: a case report

**DOI:** 10.1186/s40981-024-00719-y

**Published:** 2024-06-06

**Authors:** Satoshi Uchida, Daiki Takekawa, Koudai Kato, Kazuyoshi Hirota

**Affiliations:** 1 Department of Anesthesiology, Hirosaki University Graduate School of Medicine, 5 Zaifu-Cho, Hirosaki, 036-8562 Japan; 2 Department of Perioperative Medicine for Community Healthcare, Hirosaki University Graduate School of Medicine, 5 Zaifu-Cho, Hirosaki, 036-8562 Japan; 3 Department of Perioperative Stress Management, Hirosaki University Graduate School of Medicine, 5 Zaifu-Cho, Hirosaki, 036-8562 Japan

**Retraction Note** **:** **JA Clinical Reports 9, 42 (2023)**

**https://doi.org/10.1186/s40981-023-00634-8**

The authors have retracted this article because, subsequent to the publication of this case report, The Medical Accident Investigation Committee of Hirosaki University Hospital concluded that a different mechanism had caused the acute coronary syndrome in this patient. All authors agree with this retraction.

